# Current models broadly neglect specific needs of biodiversity conservation in protected areas under climate change

**DOI:** 10.1186/1472-6785-11-12

**Published:** 2011-05-03

**Authors:** Mungla Sieck, Pierre L Ibisch, Kirk A Moloney, Florian Jeltsch

**Affiliations:** 1 Plant Ecology and Nature Conservation, University of Potsdam, Maulbeerallee 3, D-14469 Potsdam, Germany; 2 Faculty of Forest and Environment, Eberswalde University for Sustainable Development (Univ. Appl. Sciences), Alfred-Möller-Str. 1, D-16225 Eberswalde, Germany; 3 Department of Ecology, Evolution and Organismal Biology, 253 Bessy Hall, Iowa State University, Ames IA 50011-1020, USA

## Abstract

**Background:**

Protected areas are the most common and important instrument for the conservation of biological diversity and are called for under the United Nations' *Convention on Biological Diversity*. Growing human population densities, intensified land-use, invasive species and increasing habitat fragmentation threaten ecosystems worldwide and protected areas are often the only refuge for endangered species. Climate change is posing an additional threat that may also impact ecosystems currently under protection. Therefore, it is of crucial importance to include the potential impact of climate change when designing future nature conservation strategies and implementing protected area management. This approach would go beyond reactive crisis management and, by necessity, would include anticipatory risk assessments. One avenue for doing so is being provided by simulation models that take advantage of the increase in computing capacity and performance that has occurred over the last two decades.

Here we review the literature to determine the state-of-the-art in modeling terrestrial protected areas under climate change, with the aim of evaluating and detecting trends and gaps in the current approaches being employed, as well as to provide a useful overview and guidelines for future research.

**Results:**

Most studies apply statistical, bioclimatic envelope models and focus primarily on plant species as compared to other taxa. Very few studies utilize a mechanistic, process-based approach and none examine biotic interactions like predation and competition. Important factors like land-use, habitat fragmentation, invasion and dispersal are rarely incorporated, restricting the informative value of the resulting predictions considerably.

**Conclusion:**

The general impression that emerges is that biodiversity conservation in protected areas could benefit from the application of modern modeling approaches to a greater extent than is currently reflected in the scientific literature. It is particularly true that existing models have been underutilized in testing different management options under climate change. Based on these findings we suggest a strategic framework for more effectively incorporating the impact of climate change in models exploring the effectiveness of protected areas.

## Background

Protected areas are the most common and important instrument for the conservation of biological diversity and are specifically called for under the United Nations' *Convention on Biological Diversity *[1]. Protected areas, which are developed and established to protect and maintain species, communities and ecosystems in a human-altered landscape, already cover 12% of the earth's surface [1,2]. To fulfil their task successfully, protected areas must encompass a high degree of the world's extant biological diversity and maintain a protective role over time in a dynamic landscape [3,4]. Typically, protected areas are part of a static conservation concept developed to ensure biodiversity patterns and species persistence within a given area [3,5]. This static approach is problematic given currently predicted long-term environmental changes due to a diverse array of climate-change-induced stresses [6]. In particular, it is predicted that climate change will lead to a shift in species composition within protected areas due to range-shifts and species turn-over [7-9]. The predicted changes include poleward range expansions, as well as shifts to higher elevations, and, in the case of species of the northern hemisphere, a decline of occurrence and abundance at the southern range boundaries [4,10]. If climatic alterations take place as predicted, static protected areas may not assure habitat persistence and ecosystem functioning and may not, in the long run, support all the species they were designed to protect [3,5,11].

Factors other than climate change are also expected to dynamically influence and negatively impact the efficacy of protected areas. Growing human population densities, intensified land-use, invasive species, often linked to changes in habitat heterogeneity, increasing habitat fragmentation and limited dispersal capacities are threatening ecosystems world-wide. Indeed, the effects of these factors on protected areas can be further amplified by changing climatic conditions [4,12]. Under these circumstances, protected areas, often the only viable refuge for species, are put at risk. Consequently, it is of crucial importance to incorporate a consideration of these additional factors in the development of more proactive management strategies that are based on risk assessments that go beyond reactive crisis management.

Larger protected areas, buffer zones, and connectivity between reserves have been discussed as potential strategies to counter the increasing pressure on biodiversity [11]. Even mobile protected areas that are shifted in time and space have been suggested as a buffer for climate change and increased land use [3,11]. However, the potential for evaluating and testing these approaches is often limited due to the lack of sufficient long-term data on ecosystems and environmental change [13]. One way to partly circumvent this problem is the cautious use of computer modeling, taking into account the limitations and inherent uncertainties [14-16]. Computer capacities and performance have risen significantly in the last two decades and computer modeling has been increasingly utilized in the field of conservation biology. By the 1980s, modeling studies of species range shifts using software tools, such as BIOCLIM [17] and DOMAIN [18] had already shown that species may move out of reserves due to habitat alterations and range shifts [11]. Since then, the quantity and quality of modeling tools applied in a broad range of disciplines like ecology, wildlife biology and conservation biology have increased constantly [13]. The development and improvement of climate models, such as global climate models (GCM) and regional climate models (RCM), have facilitated the incorporation of climate change projections into species models and conservation strategies [19,20]. As a consequence, several different modeling approaches have been developed and carried out to examine and assess the possible impacts of climate change on species and ecosystems [16,21-23].

Most studies modeling climate change impacts on biodiversity apply bioclimatic envelope models, also referred to as habitat models, which are purely statistical and based on a correlational approach [24]. These models capture the full ecological niche of a species or a species set based on biotic and abiotic factors. The results are often species distribution maps showing the existence of suitable habitats under current and future climates. The combined application of several bioclimatic envelope models within a single study is a recently emerging technique. Here several models are coupled with different climate scenarios in order to either determine the model with the best predictive performance or to apply a consensus approach (i.e., to provide a summary of the variation within the prediction ensemble) [25]. Ensemble forecasting produces, in general, more robust predictions and reduces uncertainty among models when incorporating climate change [26,27].

Other types of models that are being used to assess the impacts of climate change on species and ecosystems are process-based models [22]. Here we refer to an ecological model as being process-based (or mechanistic), if species performance (e.g. growth, fitness etc.) is incorporated and dynamically linked to biological and environmental factors [13]. There are different kinds of process-based models: Dynamic global vegetation models (DGVM), for example, are widely used to simulate current and future global vegetation patterns based on carbon, nutrient and water cycling [13]. Other process-based approaches focus instead on the population level and include key demographic processes in a dynamic process-based, bottom-up approach. However, these types of models, although incorporating crucial mechanistic processes, have not yet been widely applied in ecological forecasting [22].

In addition to employing purely statistical and purely process-based approaches, there has been some progress in combining the benefits of these two model types [28,29]. The so called hybrid-models, for example, include demographic and phenomenological models in simulating distributional ranges and habitat dynamics [28]. These modeling approaches and their continual improvement offer great opportunities for the researcher with respect to the integration of selected key mechanisms such as dispersal limitations or Allee-effects into statistical distribution modeling.

In this review, we examine and evaluate trends and gaps in current modeling approaches of climate change impacts on protected areas. More specifically we ask whether current modeling approaches are appropriate and sufficient to aid us in protecting species diversity within protected areas under changing climatic conditions. Focusing on terrestrial protected areas, we aim to provide a useful overview and generate guidelines for future research in order to establish adaptive and 'climate-proof' conservation strategies for protected areas.

We review the existing literature on the topic over the last twelve years (1998 - June 2010). The focus lies on the evaluation of (i) current modeling approaches and their usage in the fields of conservation biology and ecological forecasting of protected areas, (ii) the species or species groups of main interest, (iii) implementation of additional threats and key processes in the models, e.g., land-use, habitat fragmentation, invasion and dispersal, and (iv) the testing and implementation of specific management actions.

## Results and Discussion

### 1. Time of publication, geographical distribution and spatial setting

**Figure 1:**
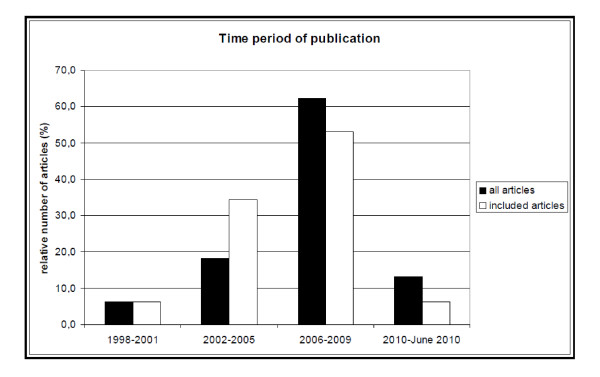
**Publication rate of articles that deal with modeling of protected areas under climate change**. Dark columns: all articles found under the applied search algorithm for the time span 1998 to June 2010; light columns: subset of articles included in this review.

Our literature search using ISI Web of Knowledge produced 394 hits. Forty three of the articles were focused on marine, aquatic or hydrologically related topics and were therefore not included. Other studies were excluded since they only discussed the potential impacts of climate change and did not simulate different scenarios or consider their implications. Many of the remaining studies simulated shifts in species distributions under changing climate, but did not explicitly focus on protected areas. In the end, 32 studies were used as they fulfilled all three criteria for inclusion as described in the methods. Despite the small body of literature, there is evidence for increasing interest in the impacts of climate change on protected areas over the last 10 years (Figure 1).

Although there is increasing interest in the topic of climate change impacts on protected areas, there is also a substantial bias in the geographical distribution of the studies published. Only two of the studies included here focused on the global network of protected areas. At the other end of the spectrum, three articles focused on individual protected areas (i.e., birds and mammals in the Arctic National Wildlife Refuge, Alaska,[30]; cacti in the Tehuacan-Cuicatlan Biosphere Reserve, Mexico,[31]; and elk in the Rocky Mountain National Park, USA,[32]). The remaining studies focused on networks of protected areas that were regional in scope.

A majority of the regional studies were focused on the African continent, more specifically Sub-Saharan Africa (Table 1). Several studies were also carried out in North America (USA and Canada), covering a large variety of ecosystems and species. In contrast, South/Central America and Europe were represented by only a few studies each (Table 1). There was just one study from Australia and none from Asia. The relatively small number of studies focusing on protected areas in Europe may also be connected to the issue of scale. Typically the spatial resolution of General Circulation Models (GCM) is rather coarse and grid cells used for these models are often bigger than many protected areas. Applying GCM results to protected area models thus requires a significant interpolation effort with reduced accuracy of model forecasts. This could be of particular relevance for Europe where protected areas are generally smaller in size. Also, in Europe, with its intensively transformed cultural landscapes, a static and representation-oriented conservation approach often prevails. It is clear that the acceptance of unavoidable change has been progressing only slowly among conservationists.

**Table 1:** Summary of the publications reviewed with regard to several factors.

Reference	Species type	**Species nr**.	Geograph. distribution	Spatial setting	Temporal scale	Methods	Land use	Dispersal	Management
[33]	P	1200	Europe	pa-s	2050	s (bcm)	no	y (m)	t

[12]	P	3	Australia	pa-s	2030;2070	s (bcm)	no	no	d

[10]	P; M	213	USA	8	na	s & p-b (DGVM)	no	no	no

[34]	P;M;B;A;F;L;MO;BR	131	USA	pa-s	2011-2040; 2061-2090	s (bcm)	no	y (sm)	t

[26]	B	50	SA; Swaziland; Lesotho	pa-s	2070-2080; 2090-2100	s (bcm)	y (cm)	y (m)	d

[72]	P	na	USA	pa-s	na	s (bcm)	no	no	d

[30]	M; B	11	USA	1	2040	s (bcm)	y (cm)	no	d

[73]	P	327	SA (CFR)	pa-s	2050	s (bcm)	y (cm)	y (m)	d

[35]	P;B;M	1695	Europe; CFR; Mexico	pa-s	2050	s (bcm)	y (m)	y (m)	t

[74]	B	1608	Sub-Saharan-Africa	803	2025; 2055; 2085	s (bcm)	no	no	d

[75]	P	na	Switzerland	109	na	s (bcm)	no	no	d

[76]	na	na	global	pa-s	2070-2100	s (MCE)	y (m)	no	no

[27]	P	1	Austria	214	2050, 2080	s (bcm)	y (m)	no	d

[2]	B;M;A;R;	25711	global	30965	2000;2050;2100	s (MA)	y (cm)	no	d

[59]	P	na	Canada	2979	na	p-b (GVM)	no	no	d

[77]	P	8	Mexico	69	2025; 2065	s (bcm)	no	no	d

[78]	B	38	Brazil	73 & 1000	2050	s (bcm)	no	y (m)	d

[7]	P	5197	Africa	IPCs & IPAs*	2025;2055;2085	s (bcm)	no	no	d

[50]	P	282	SA (CFR)	pa-s	2010-2050	s (bcm)	y (m)	y (sm)	t

[54]	P	301	SA (CFR)	pa-s	2000;2050	s (bcm)	no	y (sm)	d

[58]	P	na	Canada	39	na	p-b (GVM)	no	no	d

[31]	P	20	Mexico	1	2030-2060;2060-2100	s (bcm)	no	no	d

[79]	P	29	Mexico	pa-s	2050	s (bcm)	no	no	d

[60]	P	159	Namibia	12	2050;2080	s (bcm) & p-b (DGVM)	no	y (m)	d

[80}	P	31	Scotland	3	2080	s (bcm)	no	no	no

[81]	hlz	na	Africa	pa-s	2065;2100	s (hlz)	y (indirect in hlz)	no	no

[82]	hlz	na	Mexico	33	na	s (hlz)	y (indirect in hlz)	no	d

[4]	B;I;A;M	9	Europe	pa-s	2020;2050	s (bcm)	y (m)	y (sm)	t

[57]	P	na	Brazil	pa-s	na	s & p-b	y (m)	no	d

[32]	M	1	USA	1	2025	s & pop-dyn.	no	no	d

[8]	P	316	SA (CFR)	pa-s	2050	s (bcm)	y (m)	y (sm)	t

[9]	B	12	Sub-Saharan-Africa	pa-s	2055	s (bcm)	y (m)	no	d

Abbreviations:

P: Plants; M: Mammals; B: Birds; I: Insects; R: Reptiles; A: Amphibiens; F: Fungi; L: Lichen; MO: Molluscs; BR: Bryophytes

na: not applicable (consider different units than species number, e.g. biome change);

hlz: holdrigde life zone; pa-s: already existing protected area system

s: statistical; p-b: process-based; bcm: bioclimatic modeling; MA: Millenium Ecosystem Assessment; pop-dyn.: population dynamics; MCE: mediterranean climate extent;

GVM: global vegetation model; DGVM: dynamic global vegetation model;

Land use: y (m): land use modeled; y (cm): land use changes modeled

Dispersal: y (m): dispersal modeled as full or null dispersal; y (sm): species-specific dispersal characteristics modeled;

Management: t: tested; d: discussed

* IPC: Important Plant Cell, IPA: Important Plant Area

The geographical distribution of studies may also be a reflection of where the research groups applying these approaches are currently working. However, the large number of studies about the impact of climate change on protected areas in Africa may also be related to the data available in that region. In particular, the Proteaceae in the Cape Floristic Region are well studied. Abundance as well as occurrence data are easily accessible on the internet for a large number of species in that area http://protea.worldonline.co.za. As a result, five modeling studies fulfilling the criteria of this review are focused on the Cape Floristic Region (CFR) (Table 1).

Such a restricted geographic focus could be seen as biased and raise the concern that other regions containing biodiversity hotspots and rare species might be overlooked, providing little or no assessment about the potential impact of climate change on their status. However, the situation for the Cape Floristic Region indicates that a good, accessible data base may facilitate and help improve modeling approaches and should thus be seen as an encouraging example.

Given the typically large scales associated with climatic change and species distributions, it seems reasonable to focus on more than a single protected area. However, in order to understand mechanisms of local species and community dynamics it is necessary to also examine specific protected areas on a small scale. In this review we found only a small number of area-specific studies indicating that there is, as of yet, little model-based research into climate-adaptive conservation and management options at the small scale of individual protected areas.

While 31 of the studies examined already existing or proposed protected areas or area networks, one article concentrated on the concepts and theory underlying the selection of protected areas. This study [33] examined plants in Europe and compared six different, reserve-selection methods under climate change. Their results clearly highlight the importance of incorporating potential climate change in future decisions about protected area site locations. They also demonstrated the necessity of applying a multi-method approach in order to have the most comprehensive overview and to account for a range of uncertainties.

### 2. Number and type of species

Plant species were the focus of two thirds of the articles (n = 21; 65.6%) (Table 1); three of these studies also modeled other species [10,34,35]. Birds (n = 9; 28.1%), mammals (n = 7; 21.9%) and amphibians (n = 3; 9.4%) were also included in several studies. A multi-species study [2], modeling large numbers of mammals, birds, amphibians and reptiles (overall 25711 species), was the only one incorporating reptiles. The latter study was designed to quantify the exposure of the global reserve network to potential climate and land-use change based on the *Millennium Ecosystem Assessment*. The only study including an insect species (i.e., the large heath butterfly (*Coenonympha tullia*)) also utilized a multi-species approach, and included mammals, birds and amphibians (overall nine different species) [4]. In general, it is not surprising that there is a focus on plant species. In comparison to animal species, data collection on the geographical distributions, habitat requirements and population dynamics of plants is easier, due to their sessile life form.

The general observation that insects and reptiles are neglected in modeling studies of protected areas is notable, since several studies do exist that model shifts in the geographical distribution of butterflies (e.g., [36]) and reptiles [37,38] under climate change. Although these studies do not focus on protected areas, they do indicate the existence of sufficient data for modeling these taxa. Why this expertise and knowledge has not yet been transformed and included in studies on protected areas under climate change remains unclear.

There are additional examples of existing data and modeling expertise that, although relevant, have not been utilized in the study of protected areas. A study by Virkkala et al. [39] is a case-in-point. Their study focuses on northern boreal land birds, which are endangered because the Arctic Ocean presents a natural barrier to their distribution and inhibits any northward range shifts in response to climate change. This limitation is comparable to species living within a protected area surrounded by fragmented and unsuitable habitat. In a second example, Wichmann et al. [40] examine population dynamics and extinction risks of a long lived raptor in an arid environment under climate change using an individual orientated modeling approach. The default parameter set for this model refers to the area of the ''Kgalagadi Transfrontier Park'' situated in the arid savanna at the north-western tip of the Republic of South Africa and south eastern Botswana. This example shows the usefulness of an individual based approach in order to study the impact of climate change on species that, at least partly, depend on protected areas. Both of these examples illustrate how existing approaches might be modified to aid in the understanding of climate change impacts on protected areas.

A general trend towards a multi-species modeling approach is confirmed by most of the remaining articles (n = 22; 68,75%) (Table 1). Almost half of the publications work on more than 50 species. Several studies focus on species groups, modeling even more than 1000 species, most of them plants. Studies on animal species appear to be less extensive in the number of species modeled.

The existence of many multi-species modeling studies shows the increase in computer expertise and capacity. Although a multi-species approach provides more information about the impacts of climate change on a specific region and a specific species group than a single-species approach, it still does not account for inter- and intraspecific interactions. Mechanisms like competition and predation are crucial for understanding species and ecosystem dynamics and can be considerably affected by climate change [41]. However, none of the reviewed publications include these mechanisms, demonstrating the need for more comprehensive data and improved modeling approaches.

### 3. Additional threats

Habitat fragmentation and loss, land use and related changes in habitat heterogeneity, quality and availability, e.g. triggered by changed fire regimes, soil characteristics or biological invasions are crucial challenges for the effectiveness of protected areas and all of these threats might be affected by changing climate [5,23,42,43]. This specifically holds true when protected species also rely on surrounding landscapes and buffer zones that are more prone to human impact [44]. However, only one modeling study on climate change impacts on protected areas [4] explicitly includes habitat fragmentation as an additional factor. In contrast, 14 articles examine land use in addition to climate change, but only nine of those studies incorporate different land-use scenarios into climate change projections (Table 1). This is problematic since land-users are likely to change their practices in response to changing climatic conditions [45].

Only two articles were found that considered invasion risk to protected areas [12,27]. This is surprising as invasive species are known to be of great danger to native flora and fauna and many examples exist of considerable and severe changes in ecosystems due to non-native species [46-48]. It is also known that protected areas are not immune against infestation, as such, and therefore this is a very important point to be considered in future adaptive management of protected areas under climate change. This is especially important for protected areas within regions particularly prone and sensitive to climate change like the polar regions [48].

### 4. Dispersal

Most studies did not include any dispersal limitations. Only eleven articles explicitly explored dispersal effects and only four of them modeled species-specific dispersal capabilities (e.g. [8]). The other seven articles set dispersal as unrestricted and "tested" against no dispersal, an unrealistic assumption (Table 1).

Species dispersal is a key-process for the survival and persistence of species. This becomes even more important in a time of climate change where habitat distributions are likely to shift [49]. Therefore the ability of a species to migrate and disperse to new climatic space is critical for its long term existence. Dispersal is strongly affected by landscape conditions and therefore intensified land-use and increased habitat fragmentation are likely to lead to decreased dispersal possibilities within protected areas and the surrounding matrix. It is thus crucial to assess the combined impacts of land-use, habitat fragmentation and climate change on dispersal and include the results of this evaluation into risk assessments of protected areas under climate change [4].

### 5. Management

It is especially important to evaluate the consequences of existing and future management strategies in the face of climate change. This, for example, includes an evaluation of the effectiveness of corridors facilitating dispersal in protected area networks or the success of implementing additional conservation areas. However, only a few studies (n = 6) actually implement and test different management strategies within their modeling frameworks (Table 1). Twenty-two studies discuss some management options, but do not evaluate their effectiveness under changing climate conditions and the remaining four studies do not mention management at all. Given the fact that all reviewed studies find a negative effect of climate change on species persistence in protected areas, it is striking that only few of them explicitly use the potential of their models to evaluate alternative management scenarios. Again, an exception is provided by the intensively studied Cape Floristic Region. This area can be seen as an example of how available data can be used to provide valuable management recommendations under climate change [8,35,50].

### 6. Modeling approaches

An examination of the modeling approaches applied in the reviewed literature shows a distinct preference towards statistical methods (Table 1). We found 27 studies based on a statistical modeling approach, including 22 studies using bioclimatic modeling to answer the question of the impact of climate change on species distributions and protected areas. Studies applying a bioclimatic modeling approach compare current and future species distributions with the current locations of protected areas, as a means of assessing the protection status of species now and in the future. This approach gives a valuable, first impression of the potential of current protected areas to accommodate potential range shifts of species under climate change. However, this purely correlational methodology has several important shortcomings that have been frequently criticized (e.g. [13,19,51,52]). The main points of criticism for classical bioclimatic models, which are also valid in the context of protected areas, are the missing elements of (i) biotic interactions (e.g. [41,52,53]), (ii) dispersal limitations (e.g. [23,49,50,54]), and (iii) possible adaptations (e.g. evolutionary or behavioural) (e.g. [55,56]). Furthermore, bioclimatic models assume that species are at equilibrium and thus typically do not consider transient dynamics, even though non-equilibrium conditions are highly relevant in environments undergoing climate change [13]. An additional aspect of concern in applying bioclimatic modeling to protected areas is the spatial scale [51,53]. Currently, bioclimatic models coupled with climate change scenarios are too coarse in resolution to accurately project shifts of species distributions on the local scale. This needs to be considered and addressed specifically when evaluating climate change impacts on the scale of specific protected areas [53].

Despite the described limitations, bioclimatic models may still provide a good starting point and guide for research on climate change impacts on protected areas, due to the insufficient data currently available on dynamic mechanisms and processes. However, these models still need to be viewed and evaluated with caution (compare [41,52]).

Only five studies tackle the problem of climate change impacts on biodiversity in protected areas by applying a process-based approach. One study, [57], examines the buffering effects of protected areas on climate-tipping points in the tropical forest of Brazil, choosing a statistical approach for the vegetation (LEAF-2 submodel) and a process-based model for the hydrology (RAMS model). The other four studies use a process-based approach to model vegetation dynamics. Two works, [58] and [59], study vegetation (biome) changes within protected areas in Canada, applying global vegetation models (BIOME3 and MAPSS). The three remaining studies carrying out process-based analyses incorporate a combination of statistical and process-based methods. Burns et al. [10] and Thuiller et al. [60] apply dynamic global vegetation models (DGVM). The former [10] uses the results of a DGVM to model range shifts and species turnover of mammals within protected areas, strictly as a function of expected vegetation shifts due to predicted climate change. In comparison, Thuiller et al. [60] applies a DGVM to examine the potential impacts of climate change on vegetation structure and ecosystem functioning, whereas statistical niche-based models (NBMs) are used to assess the sensitivity of plant species to climate change.

In contrast to the species-specific bioclimatic models, DGVMs are generalized by the use of plant functional types (PFTs). This generalization allows for an assessment of the global (if this is of interest for the study) distribution of vegetation patterns and their possible range shifts due to climate change. However, DGVMs necessarily provide only a coarse representation of plant species diversity, which is often not sufficient for nature conservation [13,16]. The localized nature of most protected areas further reduces the suitability of this model type for the assessment of specific protected areas or networks under climate change (but see [61]). Another shortcoming of DGVMs is the exclusion of demographic population processes, as well as of evolutionary changes and dispersal mechanisms (see above).

There are several reasons that can explain the small number of process-based studies. First of all, the general lack of data on processes and mechanisms as well as the challenge of dealing with inherent uncertainty limits our ability to develop accurate, process-based models [16]. This specifically holds if we have to consider interacting systems and dynamically changing conditions. Interestingly, all of the reviewed process-based models focus only on plants and none incorporate population dynamics or inter- and intraspecific interactions. It is also surprising, that no study was found applying a hybrid-model type, i.e., combining advanced statistical and process-based methods, although this approach is increasingly applied in other fields of nature conservation and global change biology (e.g. [28]). Also systematic comparisons of different modeling approaches for the same conservation targets or areas, as is being increasingly used for predictions of species range shifts (e.g. [62]), would be desirable in the context of studies on protected areas. However, given the urgent need to include feedbacks and transient dynamics, we strongly recommend the intensification of the development of process-driven models for the conservation of protected areas under climate change. Clearly this also has to include information from well-designed data collection and experimental studies, which focus on elucidating mechanisms, rather than only describing patterns of occurrence and abundance. We are aware that such empirical studies in conservation areas are typically restricted by logistics (e.g. in remote areas), technical capacity or financial constraints. In certain contexts, it must be also carefully evaluated whether the gathering of new data is more relevant than actual protection activities targeting threat abatement. Research itself can even cause additional environmental harm, which of course has to be avoided. In such cases comparative approaches with other areas and more general modeling studies can be recommended. In either case, improved model parameterization and a strong emphasis on model validation methods and uncertainty analyses need to be employed [14,16,21,63].

## Conclusion

The small number of publications found for this review indicates that protected areas under climate change are still a widely neglected field of research in the modeling community. Furthermore, the limitations of currently applied modeling approaches, the high number of missing but crucial factors (e.g. land-use changes related to climate change, fragmentation, dispersal limitations, species interactions, transient dynamics etc.) and the focus on only a small number of taxa, revealed by this review, show the necessity for considerable model improvement, if we want to assess and counter climate change impacts on protected areas effectively.

Being fully aware that models will never be able to recreate reality, models should be seen primarily as an additional, but important tool for proactive and risk-oriented decision making in protected areas. However, in the absence of sound data and access to suitable modeling technologies, a precautionary risk management approach should be recommended. This would be based on the best knowledge available and should present a no or low-regret option for action. Monitoring and expert-based assessments are complimentary approaches to knowledge generation. Whenever modeling is feasible, we propose the following four-level, strategic, modeling framework:

### 1. Bioclimatic models

State-of-the-art, multi-species, bioclimatic models will remain useful as a first step in a broader modeling framework designed to evaluate the potential distributions of critical species in protected areas or protected area networks. This holds in spite of the described limitations.

### 2. Hybrid-models

Key mechanistic elements (e.g., species-specific dispersal models, Allee-effects, etc.) should be integrated into bioclimatic models for as many species as possible, in order to overcome some of the current limitations. The species and mechanisms to include in any particular model will differ depending upon the protected areas being considered and will have to be decided on a case-specific basis. This type of model has already been successfully applied to conservation related questions (e. g. [28,29,64]) and could be easily transferred to modeling protected areas under climate change.

### 3. Process-based models

Fully developed, process-based models should integrate key mechanisms into their structure with regard to assumed climate change impacts, possibly including ecophysiological or demographic processes, dispersal in the given landscape setting, species interactions, and adaptations. In any case, anthropogenic impacts on the system and the surrounding landscape (e.g. land use adaptations to climate change) should also be explicitly considered. The availability of process-based models for a selected subset of species will improve the general mechanistic understanding of possible climate change impacts on protected areas and will allow for a quantification of extinction risks for key species under different scenarios.

In most protected areas there are, in fact, focal species that have been selected for more detailed research and monitoring, e.g. because of their conservation status, because of their ecological relevance, because they are good representatives for a larger subset of species or because they are good indicators for certain habitat conditions [5,65]. The availability of data for this subset of species offers the opportunity to develop more detailed process-based models.

### 4. Testing of conservation management options

A major advantage of developing suitable models is the ability to systematically explore the likely impacts of alternative climates and also alternative management scenarios. We strongly recommend putting more emphasis on the application of already existing and to-be-developed models as tools for decision making and management support. Also steps 1-3 of the proposed modeling strategy should make full use of this potential and explicitly evaluate the consequences of current and potential alternative management plans for biodiversity conservation in protected areas under climate change. This will not only allow the detection of the limitations of certain management actions, but will also help to identify cost-effective management options It will also strengthen the bridge between more theoretical approaches (modeling) and the more practical and applied field of conservation biology.

Clearly, the modeling framework outlined above also requires a thoroughly designed strategy for monitoring reserve systems and standardizing data collection. This should not only include the collection of parameters important for recording key processes, such as dispersal or changes through adaptation to new conditions, but should also help to identify trends in species performance providing a mechanism for testing and validation of predictive models. This combined modeling and monitoring framework will allow the continual refinement of existing modeling tools and thereby improve our ability to adapt the management of protected areas to the threats of global change.

## Methods

We restricted our literature research to peer-reviewed articles on protected areas in terrestrial systems. We focused explicitly on modeling approaches exploring or predicting climate change effects on species performance and species diversity within protected areas in order to examine the information and knowledge available on this important tool for nature conservation. We are fully aware of the extensive body of literature available for modeling species reactions under climate change, often covering whole species geographical distributions. However, much of this literature does not specifically deal with protected areas. For this reason, we concentrated on this particular field in conservation biology to determine its status. We (and others) consider the design and management of protected areas as a major challenge, but also as a major opportunity, for preserving biodiversity under climate change. Therefore, we were interested in finding out whether the number of peer-reviewed publications on protected areas under climate change represents an appropriate subset of the large amount of scientific literature available on modeling species under climate change.

In our review, we excluded studies focusing on aquatic environments (e.g. marine protected areas, MPA) or studies solely related to hydrological problems. Although similar methods are often applied in marine and aquatic environments, the drivers and factors affecting species survival and protected area effectiveness in marine and aquatic systems are often quite different from those impacting terrestrial systems [66,67]. Furthermore, it is thought that climate change will influence marine and aquatic ecosystems in very different ways compared to terrestrial ecosystems [68-71].

We are also conscious of the existence of grey literature available on the topic of nature conservation and climate change. However, for reasons of simplicity and comprehensibility and, as we concentrate on the specific aspect of modeling, we considered this type of literature as less informative in order to provide scientifically reliable and traceable conclusions. Therefore, we disregarded the grey literature all together without trying to diminish its importance in the field of conservation biology and management.

We used the ISI Web of Knowledge and applied the filter "science and technology" and searched all databases for articles published since 1998. We then applied the following search algorithm to achieve a comprehensive collection of published journal articles (ISI Web of Knowledge search):

"(protected areas AND climate change AND model*) OR (conservation areas AND climate change AND model*)". Additionally, we also searched on "(protected areas AND climate change) OR (conservation areas AND climate change)" selecting articles within the search results that included a significant modeling component but were not identified with the above-mentioned search criteria. Some other search terms such as "global change", "nature reserves" and "national parks" were also tested, but did not lead to new articles fitting the aim of this review. We further limited the resulting database by restricting the review to articles that explicitly focused on terrestrial protected areas (in contrast to just mentioning them in the discussion or conclusion), and also explicitly included future projections related to climate change scenarios.

Thirty-two peer-reviewed articles were found fulfilling the above-mentioned criteria and were included in the analysis. We do not claim to have found the complete list of relevant publications, but we do have a representative overview of the articles on the topic of terrestrial protected areas and climate change utilizing modeling approaches.

The publications were analyzed with respect to different categories in order to detect trends and gaps in this field. The following components were of particular interest:

### 1. Time of Publication, geographical distribution and spatial setting

We were interested in the time of publication, geographical distribution and the spatial setting of the protected areas studied. Therefore, we examined the year of publication of the reviewed articles. We divided the 12 year time span into 4 periods (1998-2001, 2002-2005, 2006-2009, 2010-June 2010). To assess the geographical distribution we investigated the global allocation (continent, country) of the protected areas studied in the reviewed literature. We also examined the spatial setting (number of protected areas) analyzed in each study. Here we specifically looked at whether the model was applied to a single case study, exploring possible climate change impacts on the local scale of the protected area or, in contrast, investigated networks of protected areas on a regional or even global scale. We further distinguished whether studies dealt with real or hypothetical protected areas.

### 2. Type and numbers of species

We examined the studies with regard to the type of species they focused on. Therefore, we differentiated among plants, mammals, birds, reptiles, amphibians, insects, fungi, molluscs, and bryophytes. In addition we evaluated whether the publications applied a single- or multi-species approach. If a multi-species modeling effort was conducted, we assessed the number species included in the study.

### 3. Additional threats

In order to achieve an overview of model complexity with regard to possible threats to protected areas, we examined whether land-use, habitat fragmentation, and/or invasion was included in the study. For land-use and habitat fragmentation we distinguished between "modeled change" (mc) and "modeled static" (ms). We assigned the category "modeled change" if changes in land-use over time were explicitly incorporated in future projections, and "modeled static" if land use was simulated in future projections but did not change over time.

We further evaluated the incorporation of invasive species into the model. Therefore we examined whether invasion was part of the research question and a factor implemented in the model.

### 4. Dispersal

If dispersal was included in the study we differentiated between "modeled" (m) and "specifically modeled" (sm). We used the category "specifically modeled" when species-specific dispersal characteristics were included, e.g. in the form of specific dispersal kernels. Under "modeled" we categorized all publications that only distinguished between either unlimited dispersal (i.e. no dispersal limitations) or no dispersal.

### 5. Management

To assess the information for possible management implications provided by the literature, we evaluated the publications with regard to management strategies. We distinguished between "tested" (t) (different management strategies were included in the simulations) and "discussed" (d) (different management strategies were discussed but were not explicitly included in the simulations).

### 6. Modeling approaches

We compared different approaches with respect to statistical vs. process-based methods. For this category we defined "process-based" models as those that incorporated mechanistic and dynamic modeling approaches and "statistical" models as those that were correlational and purely descriptive. We additionally considered all publications as bioclimatic modeling (bcm) if they carried out a species distribution model correlated to environmental/climatic factors.

## Authors' contributions

MS designed and conducted the literature search as well as the analysis of the found articles. PLI gave insight into the field of nature conservation, especially from the applied point of view. KAM and FJ both provided input of their extensive knowledge of ecological modeling. All authors assisted in revising the manuscript and read and approved the final manuscript.
